# Construction and Validation of an Image Discrimination Algorithm to Discriminate Necrosis from Wounds in Pressure Ulcers

**DOI:** 10.3390/jcm12062194

**Published:** 2023-03-12

**Authors:** Shunsuke Sakakibara, Akira Takekawa, Chikara Takekawa, Satoshi Nagai, Hiroto Terashi

**Affiliations:** 1 Department of Plastic Surgery, Kobe University Graduate School of Medicine, Kobe 650-0017, Japan; poti7954@gmail.com (A.T.); chi.takekawa@gmail.com (C.T.); terashi@med.kobe-u.ac.jp (H.T.); 2 Graduate School of Human Development and Environment, Kobe University, Kobe 657-8501, Japan; drgero@kdn.biglobe.ne.jp

**Keywords:** artificial intelligence, digital application, wound care, decubitus ulcer, necrotic tissue

## Abstract

Artificial intelligence (AI) in medical care can raise diagnosis accuracy and improve its uniformity. This study developed a diagnostic imaging system for chronic wounds that can be used in medically underpopulated areas. The image identification algorithm searches for patterns and makes decisions based on information obtained from pixels rather than images. Images of 50 patients with pressure sores treated at Kobe University Hospital were examined. The algorithm determined the presence of necrosis with a significant difference (*p* = 3.39 × 10^−5^). A threshold value was created with a luminance difference of 50 for the group with necrosis of 5% or more black pixels. In the no-necrosis group with less than 5% black pixels, the threshold value was created with a brightness difference of 100. The “shallow wounds” were distributed below 100, whereas the “deep wounds” were distributed above 100. When the algorithm was applied to 24 images of 23 new cases, there was 100% agreement between the specialist and the algorithm regarding the presence of necrotic tissue and wound depth evaluation. The algorithm identifies the necrotic tissue and wound depth without requiring a large amount of data, making it suitable for application to future AI diagnosis systems for chronic wounds.

## 1. Introduction

Medical care relies on diagnoses made by medically educated physicians based on their own experiences. This experience in clinical education has a specific degree of latitude and only sometimes leads to consistent diagnostic results for the same case. Additionally, in medically underpopulated areas, specialists in each field are often absent. This situation results in a bias in the medical care quality in the region. In response, online telemedicine has been introduced. This online medical care (telehealth) system was also effective during the COVID-19 epidemic [1,2,3,4]. However, the basis of online medical care requires the physician to be on the other side of the network. In contrast, a medical system using artificial intelligence (AI) is also considered to address these issues. One of the critical roles that AI can play in medicine is to raise the adequacy rate of diagnosis and make it more uniform.

AI has been introduced and put into practical use in the United States, primarily in radiological imaging [5,6]. This method utilizes deep learning, where the AI is trained based on supervised images. In addition, AI has developed a diagnostic system for wounds. Pressure ulcers are classified into Stage 1 to Stage 4 according to the depth of the damage (EPUAP). In Stage 1, the damage is superficial and likely reversible. In Stage 2, damage to the epidermis and dermis is observed but does not extend to the subcutaneous fat, whereas damage that reaches the subcutaneous fat is classified as Stage 3. When it reaches deeper muscles, tendons, bones, and so on, it is defined as Stage 4. In Stage 1 and Stage 2, no specialized wound treatment is required, as tissue damage is shallow and localized. Improvements in environmental factors that caused wound formation are the main focus of treatment. However, appropriate wound management, including proper debridement of necrotic tissue, is required in Stage 3 and Stage 4 cases. In particular, in Stage 4 cases, there is a possibility that the infection has spread to the subcutaneous tissue, and immediate intervention by a wound specialist is desirable. However, there is a shortage of personnel who can make these judgments in medically underserved areas and home care settings. Therefore, the development of a wound assessment system using AI is desired. The ultimate goal is for AI to stage pressure ulcers and propose appropriate treatment methods. However, the usefulness of AI is low for Stage 1 or Stage 2, which do not necessarily require special treatment. On the other hand, it is important to not miss cases requiring urgent attention in Stages 3 or 4 and to avoid incorrect treatments. Therefore, AI proposals are crucial. However, deep learning requires a large amount of image data. Thus, the development difficulty differs significantly between radiological images with unlimited teacher images and wound images with limited sources. In the field of wounds, systems have been developed using machine learning (ML) or deep learning (DL) to evaluate necrotic tissue, granulation tissue, slough, and so on. Many of these systems use tens of thousands to hundreds of thousands of training images, which are amplified images, but the basic images used are only a few tens to a few hundred. For example, Veredas et al. used 113 basic images to create approximately 16,000 images [7], and Zahia et al. used 22 basic images to create approximately 380,000 images [8]. On the other hand, Chang et al. used approximately 2800 basic images without amplification to compare five popular AI systems and constructed a system with a high degree of accuracy [9].

In building a wound diagnosis system using deep learning, many systems first perform wound segmentation, followed by wound measurement and tissue classification [7,8,10,11]. In studies using support vector machine (SVM) as part of machine learning, wound segmentation is also performed, and color correction is applied [12,13].

Instead of building a system to determine the stage of pressure ulcers with high accuracy using small data, we aimed to develop a system that evaluates individual items for assessing the condition of pressure ulcers and combines the data obtained from each item to perform the final wound evaluation. That is, we created a simple system that does not require wound segmentation by focusing only on the evaluation of necrotic tissue in the wound.

This study used standard Japanese decubitus ulcer images collected during medical treatment at the Department of Plastic and Reconstructive Surgery, Kobe University Hospital. We constructed an image identification algorithm to identify the presence or absence of necrosis, type of necrosis (black or white necrosis), and depth of the wound. The constructed model was also used to verify the correctness of the algorithm by matching the identification results with the physician’s judgment using images of a different case than the previous case.

## 2. Materials and Methods

### 2.1. Image Collection

In conducting this study, approval for clinical images was obtained from the Kobe University Medical Ethics Committee. Clinical images of patients with bedsores treated at the Department of Plastic and Reconstructive Surgery, Kobe University Hospital, were analyzed. Clinical images have been captured and stored in the past. Compact digital cameras were used for photography; however, a constant model was not employed. The method and environment in which the photographs were taken were not consistent. Of the images captured, 50 were randomly selected. Except for cropping the images such that the wound area was approximately 50% of each image, no other processing or manipulation was performed. Of these 50 cases, 27 were used as teacher images for the development of the system, and the remaining 23 cases (24 sites) were utilized to validate the developed system.

### 2.2. Physician Determination of Necrosis

Two plastic surgeons with experience in wound care determined the presence of necrosis and wound depth from images. First, the two participants were asked to refer to and evaluate the images individually. When there were differences in the evaluation, we discussed and reached a consensus. However, because the evaluation was the same in all 27 images, we used this answer as an exemplary answer in image identification.

In pressure ulcers, necrotic tissue changes color from black to white depending on whether it is dry or moist. To identify the color tones, both black and white are used as the endpoints of the color range, and both states, which are recognized as being far apart, must be recognized as necrotic tissue. Here, necrotic tissue that has dried and turned black is defined as “black necrosis”, and necrotic tissue that is relatively moist and appears white is defined as “white necrosis”.

For the presence of necrosis, only the presence of areas of necrosis was an evaluation item; black, white, or other states of necrosis were not listed as evaluation items. The Fibrin membrane and biofilm of the slough, which resemble white necrosis, were distinguished from necrosis.

For “wound depth“, we defined “superficial wounds“ as wounds that remain in the superficial dermis layer and “deep wounds“ as wounds that extend from the mid-dermis to the fat layer. Wounds replaced by granulation were classified as “deep“. If the image analysis output included some of the worst information in the image (presence of necrosis, deep wound), it was used to evaluate the case.

### 2.3. Determining the Presence of Necrosis Using Color Pixels

Trimming was performed on the images of the 27 cases to reduce the computational complexity of the algorithm. The cropped image was assumed to be a square, not a rectangle, and the length of one side of the image was assumed to be given by $\frac{long\;diameter\;+\;short\;diameter}{2}$ × 1.25 of a pressure sore area with long and short diameters. This approach set the area of the pressure ulcer to be approximately 50% of the image. The cropped image was then resized to 200 pixels per side (200 × 200 = 40 k pixels per image), and this image was used to identify necrosis. Black and white necrosis exist, but both should be recognized as necrosis [14].

The color depth was set as red, green, and blue (RGB) with eight bits. Therefore, 256 levels (0–255) of brightness were in each spectrum per pixel. For each of the RGB colors, the color range was set as an absolute value. The value $\left\{\left(maximum\;value–minimum\;value\right)\times 0.36+minimum\;value\right\}$ was set as the threshold value, and a black pixel was defined when R, G, and B were below this threshold value. In contrast, the average value of the color range for each of R, G, and B in the 40 k pixels that constitute the image was calculated and used as the threshold value. Next, for each pixel, when the R and G color ranges were more significant than the threshold value, the R color range was more significant than B, and the G color range was greater than B; this pixel was defined as a white pixel.

The percentage of black pixels in the image (%) was then defined as black pixels (%). Similarly, the percentage of white pixels in the entire image was defined as white pixels (%). This white pixel included the skin without pressure ulcers. These numbers were plotted on a graph with black pixels (%) on the vertical axis and white pixels (%) on the horizontal axis.

### 2.4. Construction of an Algorithm for Determining Scratch Depth Using Images

The image was converted to 50 × 50 pixels (2.5 k pixels) using the nearest neighbor method to remove small bumps and observe significant changes [15]. In addition, the pressure ulcer image was shifted diagonally by 45° by one pixel. The respective RGB color ranges were subtracted between this image and the original image at the pixels mapped to the same location. This difference was measured across the selected images, and the maximum absolute value of the change in luminance between R, G, and B was considered the luminance difference. The data of 27 cases were placed on a scatter plot with the black pixels (%) in the 40 k pixel image calculated in Section 2.3 on the vertical axis and the R luminance difference in the 2.5 k pixel image on the horizontal axis. The threshold values were contrasted with the physician’s judgments of wound depth on this scatterplot.

## 3. Results

### 3.1. Detection of Necrotic Tissue

#### 3.1.1. Pressure Ulcer Image Data of 27 Cases Used to Construct Discrimination Algorithm

Table 1 shows the pressure ulcer image data contents for the 27 cases used to construct the algorithm. Those judged by the physician to have necrosis were assigned a value of 1, and those judged to have no necrosis were assigned a value of 0. Similarly, 1 was assigned to those judged to have deep wounds, and 0 to those judged to have shallow wounds. Of the 27 patients, 17 were determined to have necrosis. In addition, 20 patients were determined to have deep wounds. Black (%) represents the percentage of black pixels in the pressure sore pixels, and white (%) represents the percentage of white pixels. The black/white ratio was calculated as black (%)/white (%) multiplied by 100.

#### 3.1.2. Determination of Necrosis Using White (%) and Black Pixels (%)

**Figure 1:**
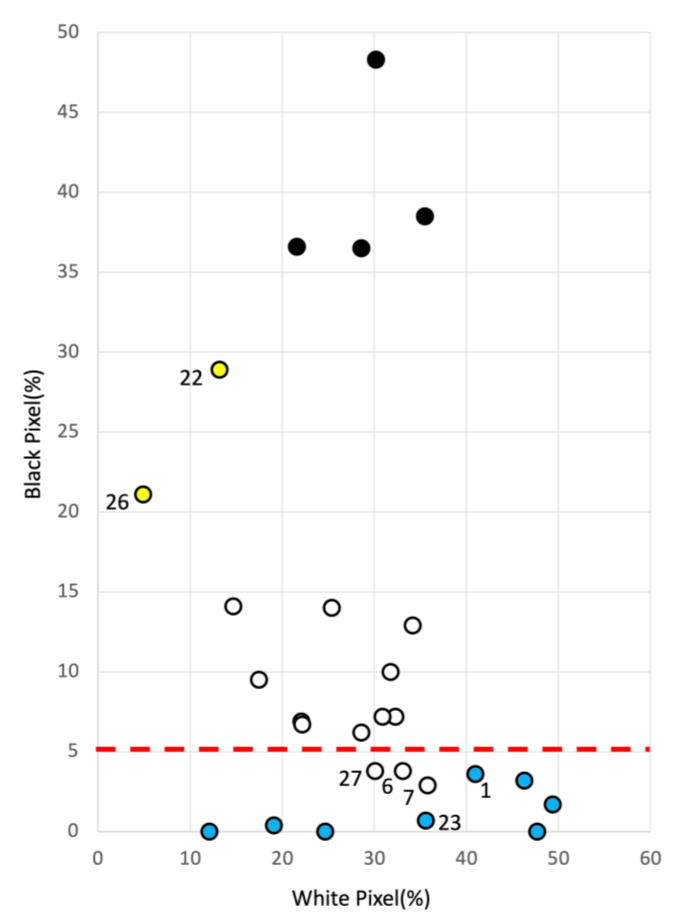
Twenty-seven cases aligned on the chart with black and white pixels. The 27 cases analyzed were divided into four groups on a plane represented by the white pixel percentage and black pixel percentage: black necrosis group (black circle), slough group (yellow circle), white necrosis group (white circle), and no necrosis group (blue circle), based on physician judgment. The patients were divided into four groups. By setting the threshold value to 5% black pixels (red dotted line), the sensitivity and specificity were 82.4% and 100%, respectively.

The 27 cases were arranged on a scatter plot with white pixels on the horizontal axis and black pixels on the vertical axis (Figure 1). The 27 cases were divided into four groups on a plane represented by the white pixel percentage and black pixel percentage: black necrosis group (black circle), slough group (yellow circle), white necrosis group (white circle), and no necrosis group (blue circle), based on physician judgment. The patients were divided into four groups. As shown in Figure 1, black necrosis was distributed in the zone with white pixel percentages between 20% and 40% and black pixel percentages between 35% and 50%. Sloughs were distributed in the zone with less than 20% white pixels and 20–30% black pixels. White necrosis was distributed in the zone with 10–40% white pixels and 5–15% black pixels, whereas some (Cases 6, 7, and 27) were distributed in the no necrosis zone with less than 5% black pixels. No necrosis was observed in zones with 0–50% white pixels and less than 5% black pixels. Table 2 summarizes the presence of necrosis. Slough zones were included in the non-necrosis group. The algorithm determined the presence of necrosis with a significant difference (*p* = 3.39 × 10^−5^), with a sensitivity of 82.4% and specificity of 100% (Table 2).

The two cases that the physician determined to be “no necrosis” (Cases 22 and 26 in Figure 1) were sloughs, but the image identification algorithm identified them as slough zones.

Three cases (Cases 6, 7, and 27 in Figure 1), which were judged by the physician to have necrosis but not by the algorithm, were judged to have “no necrosis” by image identification. This choice was made because the black pixels generated in the epidermis and dermis were measured to be low because of deep wounds. Therefore, we used $\frac{Black\;pixcels\;\left(\%\right)}{White\;pixcels\;\left(\%\right)}\times 100$ (hereafter referred to as the black-white pixel ratio) as a new index for those with less than 5% black pixels and less than 160 depth, except for Case 1, in which the wound depth presents extremely deep pockets. Thus, it was shown that an index of 8.0 or higher should be judged as “necrotic” even if the percentage of black pixels is less than 5%. This criterion considers that, when necrosis occurs, the black pixels do not drop near 0, even if they are below 5%, and the white pixels cluster around a specific value. The results of the judgment based on these criteria are shown in Table 3. In other words, using white pixels, black pixels, and the black/white pixel ratio as criteria, the algorithm could discriminate the presence of necrosis with 100% sensitivity and specificity (*p* = 1.19 × 10^−7^) (Table 3).

### 3.2. Detection of Wound Depth

**Figure 2:**
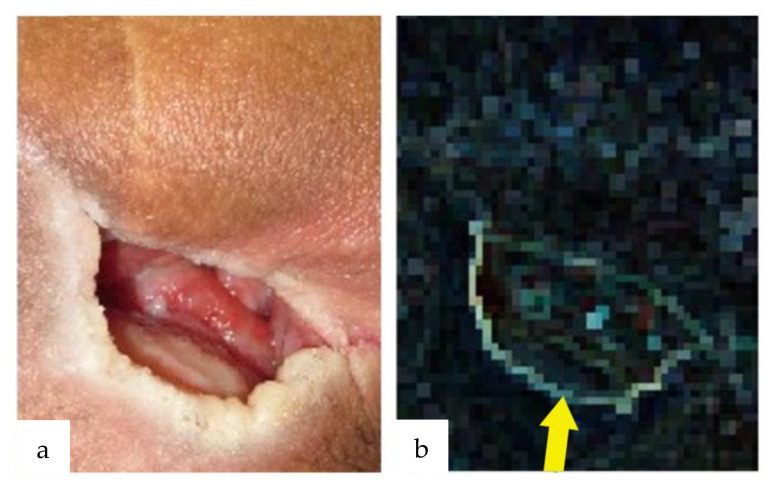
Depth of the wound depicted by the nearest neighbor method. (**a**) Decubitus ulcer with a subcutaneous pocket arising in the sciatic region. (**b**) The nearest neighbor method was used to superimpose the original image. The image shifted by 1 pixel at an angle of 45° and took the difference, resulting in a figure in which only the edge of the wound was highlighted (yellow arrow).

The nearest neighbor method was used to remove small bumps to evaluate the wound depth and observe significant changes. The results are shown in Figure 2, in which only the edges of the wound are highlighted by overlapping and differencing the original image and an image shifted by one pixel at an angle of 45°. As a result of the study using the luminance difference for each of R, G, and B, only the R component was used for the luminance difference because the case performed with the R component was the most consistent with the depth of the wound, as judged by the physician.

**Figure 3:**
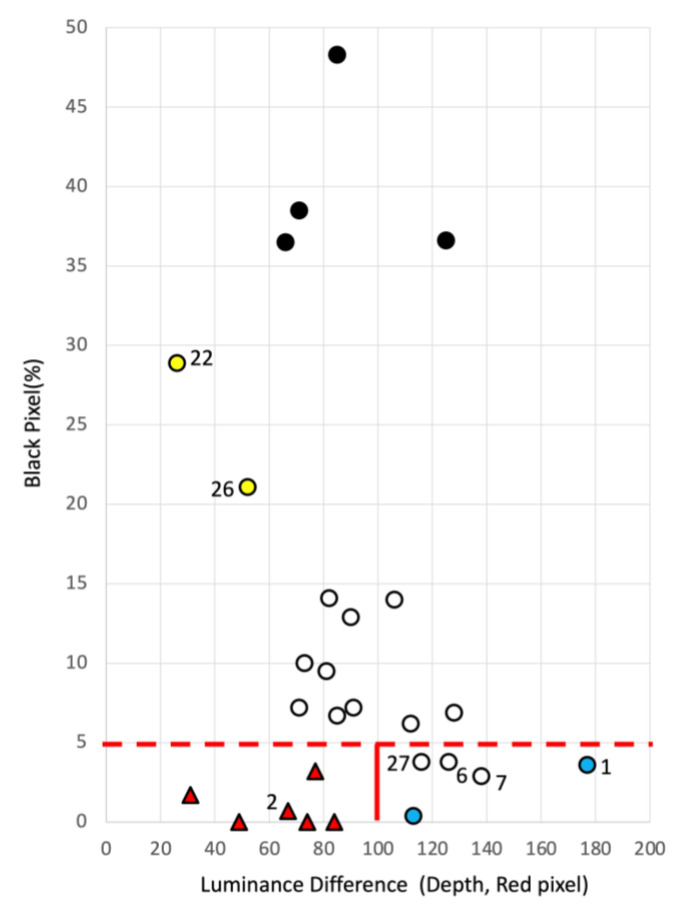
Twenty-seven cases aligned on the chart for detection of wound depth. The 27 cases were placed on a scatter plot with luminance difference on the horizontal axis and black pixels on the vertical axis. The 27 cases analyzed were divided into five groups: a shallow wound group with no necrosis (red triangle), slough group (yellow circle), black necrosis group (black circle), white necrosis group (white circle), and deep wound group with no necrosis (blue circle), according to physician judgment. In the case of the no necrosis group with less than 5% black pixels, the threshold line was created with a difference of brightness of 100, with “shallow wounds” distributed below 100 and “deep wounds” above 100. The “shallow wounds” were distributed below 100, whereas the “deep wounds” were distributed above 100. The slough group was distributed between 20% and 30% black pixels.

The values obtained from each case were placed on a scatter plot with the luminance difference on the horizontal axis and black pixels on the vertical axis for the 27 cases. Figure 3 shows these values in a graph. The 27 cases analyzed were divided into five groups: no necrosis and shallow wounds (red triangle), slough (yellow circle), black necrosis (black circle), white necrosis (white circle), and no necrosis and deep wounds (blue circle). In the case of the no necrosis group with less than 5% black pixels, the threshold value was created with a difference in brightness of 100, with “shallow” lesions distributed below 100 and “deep” lesions above 100. The “shallow wounds” were distributed below 100, whereas the “deep wounds” were distributed above 100. The slough group was distributed between 20% and 30% black pixels. Luminance differences of 40 or less were considered shallow scratches, and those exceeding 40 were considered deep scratches. The results showed that black and white necrosis were distributed between 60 and 140 in luminance difference in the group with necrosis. Still, no difference in depth between black and white necrosis was found. Therefore, in the case of necrosis, the two were not distinguished, and the wound was assumed to be deep. Table 4 lists the 27 cases judged using this criterion.

Table 4 shows that when the physician judged the wound to be “deep”, image identification also judged the wound to be “deep”. When the physician judged the wound to be “shallow”, image identification also judged the wound to be “shallow” (*p* = 1.13 × 10^−6^). The image identification also judged “shallow wounds” as “deep wounds” when the physician judged “deep wounds” (*p* = 1.13 × 10^−6^).

### 3.3. Validation of Necrosis Using 23 Cases

**Figure 4:**
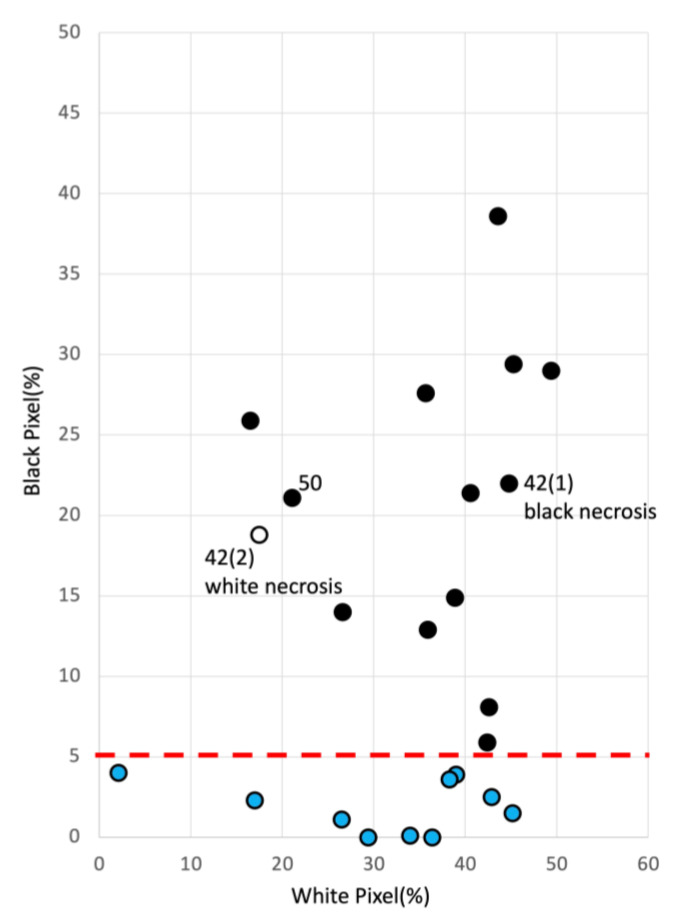
Twenty-three cases aligned on the chart with black and white pixels. Patients were divided into three groups based on physician judgment: black necrosis group (black circle), white necrosis group (white circle), and no necrosis group (blue circle). As per the algorithm depicted in Figure 1, we defined a black pixel percentage of more than 5% as necrotic and less than 5% as necrotic. We confirmed that with necrosis and without necrosis groups were separated, based on 5% black pixels.

The algorithms presented in Section 3.1 and Section 3.2 were adapted (tuned) to the information obtained from the 27 cases. Next, we tested whether this algorithm works reasonably well for other cases using 23 new cases (24 sites). Table 5 shows the pressure ulcer data for 23 cases (24 sites). All patients were evaluated by a physician for the presence of necrosis and wound depth. Figure 4 shows the presence or absence of necrosis on a plane of white and black pixels by applying the algorithm constructed for the 23 cases (24 sites). Necrosis was divided into three groups based on physician judgment: black necrosis (black circle), white necrosis (white circle), and no necrosis (blue circle). The criteria are the same as in Section 3.1 and Section 3.2. Necrotic and non-necrotic groups were separated based on 5% black pixels. Table 6 compares the physician necrosis determinations with the necrosis determined by image identification, showing 100% agreement (*p* = 5.10 × 10^−7^).

**Figure 5:**
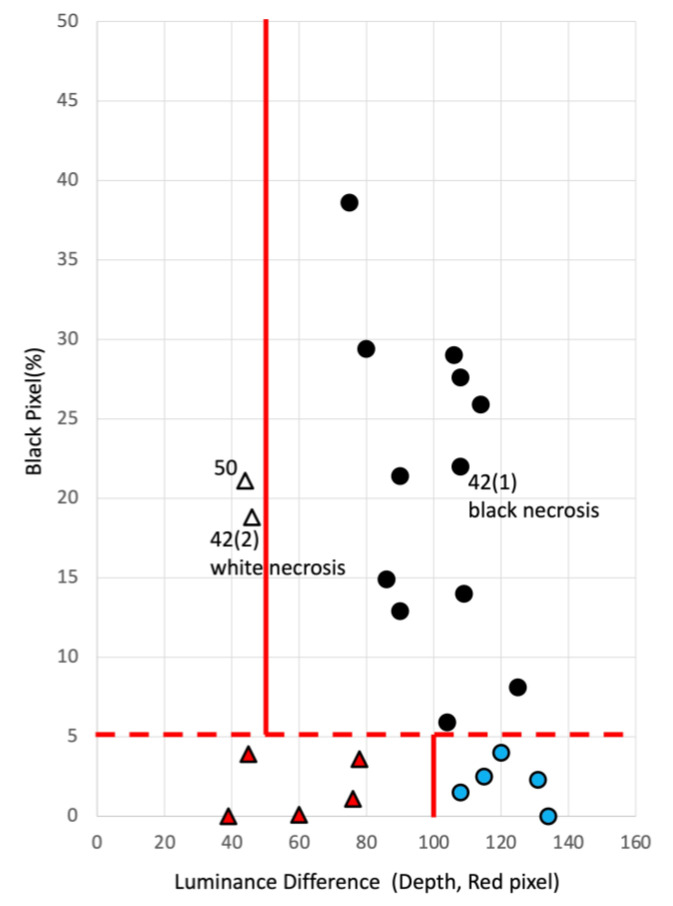
23 cases aligned on the chart for detection of wound depth. The patients were divided into four groups based on physician judgment: no necrosis and shallow wound (red triangle), necrosis and shallow wound (white triangle), necrosis and deep wound (black circle), and no necrosis and deep wound (blue circle). The presence or absence of necrosis is separated into two categories by the line of 5% black pixels on the plane of white and black pixels. All pixels above 5% are classified as “necrotic” by the algorithm. This result is consistent with the judgment of the specialist physicians.

Subsequently, we verified the wound depth. Figure 5 shows the results for wound depth. The 23 cases analyzed were divided into four groups: no necrosis and shallow wound (red triangle), necrosis and shallow wound (white triangle), necrosis and deep wound (black circle), and no necrosis and deep wound (blue circle). The presence or absence of necrosis is separated into two categories by the line of 5% black pixels on the plane of white and black pixels. All pixels above 5% are classified as “necrotic” by the algorithm. This result is consistent with the judgment of the specialists. Interestingly, for the group with necrosis with >5% black pixels, a threshold line was created with a luminance difference of 50, and white necrosis and shallow wound group were distributed below 50.

Table 7 shows the results of the comparison between the depth of the wound judged by image identification and the physician’s judgments, showing 100% agreement (*p* = 2.89 × 10^−6^). These validation results indicate that the wound evaluation algorithm established in this study accurately determines the presence or absence of necrosis and the wound depth.

## 4. Discussion

As life expectancy increases because of advances in social systems and the healthcare environment, it is clear that the number of chronic wounds, such as pressure ulcers, that commonly occur in the elderly will increase [16,17]. In contrast, wound care is only sometimes performed by experts because of the limited number of experts specializing in wound care and their uneven distribution in the region. The goals of experts in wound care are to heal wounds and shorten their duration, which are the same as the objectives of telemedicine [18]. In contrast, for non-expert healthcare providers, the critical goal of treatment is to avoid incorrect treatment. Although these objectives may differ in direction, they share the need for accurate wound assessment.

Wounds should be evaluated through a visual examination and palpation. Heat and tenderness require palpation of the three signs of infection, whereas redness is evaluated by visual examination. The presence of necrotic tissue and wound depth should be evaluated by visual examination. In other words, much information can be obtained from images.

Attempts have been made to evaluate wounds with AI, but none have thus far resulted in practical applications [11,19,20]. Howell et al. [20] traced wound and granulation areas on the images, comparing manual tracing to tracing with AI and quantitatively evaluating both. The results showed that the wound tracing by AI almost matched the manual tracing by the specialist but did not reach perfect agreement. In addition, the tracing by AI was done using existing software. Because they did not develop the software, there was no way to provide feedback on whether the AI or human tracing were accurate without knowing the insights of the software. They only used AI software and compared that software with human tracing. In contrast to their research, our image identification engine considered the judgment of a skilled specialist as true first and then took each pixel of the image and gave clinical meaning to its measurements to fit that analysis. Iizaka et al. attempted to extract the red color range to assess the conditions of pressure ulcer granulation [21]. After adjusting the color tone of the captured image using image processing software, their method extracted only the red color range using RGB filters to calculate the luminance and correlate it with the healing process. Although this method effectively evaluates changes over time in the same patient, it requires constant color correction. It does not allow for evaluating patients or wounds other than granulation tissue.

Measuring the wound area during treatment and evaluating changes in the wound area over time is an essential endpoint because it leads to the evaluation of treatment methods. However, wound size measurement does not require advanced technology. The benefits of automating wound size measurement would be to reduce the risk of infection by making non-contact measurements and free medical personnel from cumbersome processes. Identifying the wound area separately from the healthy area is the gateway to developing a wound evaluation system using AI. However, we evaluated the wound without tracing its area. By viewing the image as a collection of pixels and comparing the individual characteristics of these collections, we successfully evaluated the presence of necrotic tissue and wound depth. Because this system uses the difference in parameters from the surrounding healthy skin for its calculation, the assumption is that the photograph includes a wound to which the photographer sets the area ratio of the wound in the photograph to approximately 50%. The automatic diagnosis system we are aiming for will be useful for in-home medical care and in underpopulated areas. As we are developing a system with the expectation that the person taking the photographs in the field will be able to determine whether or not a wound is present, we believe that the problems at the photography stage will be resolved.

In this study, we did not trace the wound in the wound images. The first step in many previous studies was automatically recognizing the wound and tracing its boundaries [20,22,23,24,25,26,27,28]. However, when the AI system is used in a wound care setting, the assumption is that a medical professional (regardless of their knowledge or experience with wounds) is taking pictures and artificially determining whether a wound is present. Thus, it is possible to fit the wound area into approximately half the area of the image to be taken. We consider the entire image to be a group of pixels. The image contains both normal and wounded areas. By comparing the distribution width of this pixel information, the presence or absence of necrotic tissue and the depth of the wound were successfully determined. However, the extent of necrotic tissue was not measured, and only the deepest point of the wound was evaluated. In this respect, it can be said that the system extracts only the worst parts of the wound. However, the most critical aspect of wound management is not to neglect to address the worst parts of the wound. From this perspective, the system is necessary and sufficient.

In this study, we found that white and black necrosis could be recognized as necrosis using our algorithm. As the present evaluation focused only on the presence or absence of necrosis, no distinction was made between black and white necrosis. In contrast, in ischemic necrosis in toes, for example, black necrosis plays an important role in guiding treatment. The fact that the threshold for necrosis can be set from black and white pixels suggests that it is possible to distinguish between black and white necrosis.

This algorithm could be effective for wounds other than pressure ulcers. To determine the depth of the scratches, a threshold value of 100 was set for the R luminance difference. This threshold value was not determined by a statistical process, but rather was set based on the physician’s judgments and the distribution of each case on the graph. The set values were obtained from the data of 27 cases and, although they were not derived from a large number of cases, they were compatible with the 23 cases used for validation. The threshold value for determining the depth of the wound probably exists in the neighborhood of this value of 100. Still, a more precise value is expected as this system is implemented and more cases are added.

According to the criteria in Section 3.2, all wounds with necrosis should have been deep, but the physician’s judgment for Cases 42 (2) and 50 was that the wounds were shallow with necrosis. The same decision was made by image identification. In these two cases, necrosis was part of the wound, so the image identification did not place the wounds in the deep zone, and the wounds were identified as shallow despite necrosis. Ultimately, the judgments of the physicians and image identification system were in agreement. Figure 3 shows a very deep case (blue circle 1) that has a pocket without necrosis. Thus, based on luminance differences, the depth discrimination method makes it possible to identify the presence of pockets.

Sloughs could be evaluated using a threshold value of 5% black pixels for continuous necrosis and depth determination. This situation suggests that they are distributed in areas that are closer in color tone to the surrounding healthy skin than white necrotic areas. The classification by luminance difference 60 was used to distinguish between the depth of the necrotic wounds. Still, because only two cases were included in this study, it is impossible to determine whether this classification is correct. In the verified cases, physicians judged the wounds to be shallow despite necrotic tissue using a luminance difference of 50 as the threshold value (Cases 42 (2) and 50). In these two cases, necrotic tissue was present, but only superficially. Their similar distributions make it challenging to distinguish between these superficial necroses and sloughs. However, whether slough or superficial necrosis is present, removal of slough and necrotic tissue by debridement and response to infection is required. Even if the slough is determined to be superficial necrotic tissue in wound management, the necessary treatment details are not much different. There were no significant differences between the groups. Similarly, the slough group was positioned between the black and white necrosis groups, but this is not considered a problem in wound management, as the procedures are similar in the field treatment for the time being. In contrast, a trained physician can distinguish sloughs from necrotic tissue with the naked eye. The high water content relative to the necrotic tissue is thought to be responsible for the high white luminosity. In recent years, the prevention of slough formation has been recommended for wound management. Continued accumulation of cases may allow for more detailed classification and threshold values for sloughs.

Compared to other studies using deep learning, the number of images used in this study is relatively small. However, in each study, the same images were augmented and used, so the actual number of basic images does not differ significantly from that of this study. Because this study is not a clinical trial aimed at general treatment outcomes, we do not believe that the sample size requires to be set by power analysis. In addition, as shown in Table 2, Table 3, Table 4, Table 6 and Table 7, the results of Fisher’s exact test are close to zero, which indicates that these results are unlikely to occur by chance. Therefore, the sample size is considered sufficient to obtain these results. However, in the future, by utilizing a larger sample size, it may be possible to set thresholds in more detail and increase sensitivity and specificity accordingly.

One potential limitation of this study is that the color information extracted from the entire targeted segmented image may lead to erroneous identification if there are objects with similar color tones to necrotic tissue or granulation within the region, causing the cutoff value to be exceeded. Such objects could involve discoloration due to erythema or hemosiderin deposition in the wound surroundings, or the coloration of garments. Nevertheless, this matter might be resolved by running our developed system after the wound region is extracted. We intend to investigate this potential solution in our future research endeavors.

## 5. Conclusions

In this study, we constructed an image identification algorithm to determine the presence or absence of necrosis and the depth of the wound from bedsore images. The results showed that four factors must be identified for image identification. These factors were the percentage of black pixels from the partial image of the pressure sore, the percentage of white pixels, the ratio of the percentage of black pixels to white pixels, and the difference in value for R between the partial image and the image shifted one pixel diagonally from it. We found that once these four variables were determined, whether the necrosis was black, white necrosis, slough, or no necrosis, and whether the wound was deep or shallow was determined.

The thresholds for the four variables were determined using the pressure ulcer images of the 27 cases. The image identification results for the presence of necrosis and wound depth in the 27 cases used to determine thresholds were in 100% agreement with the judgment results of the expert physicians. The algorithm was then applied to another 24 pressure ulcer images that were not used to determine the threshold. The identification results were 100% consistent with the specialist judgments for these 23 cases (24 sites). This finding suggests that the image identification algorithm captures the essence of the discrimination between bedsore necrosis and wound depth and can accurately discriminate various bedsore images.

## Figures and Tables

**Table 1:** Data of 27 cases with decubitus ulcers.

Case	Necrosis	Depth	Black (%)	White (%)	Black/White	Red	Comment
1	0	1	3.6	41	8.8	177	Pocket
2	1	1	6.9	22.1	31.2	128	
3	1	1	9.5	17.5	54.3	81	
4	1	1	36.5	28.6	127.6	66	
5	1	1	48.3	30.2	159.9	85	
6	1	1	3.8	33.1	11.5	126	
7	1	1	2.9	35.8	8.1	138	
8	1	1	7.2	32.3	22.3	91	
9	1	1	12.9	34.2	37.7	90	
10	1	0	6.7	22.2	30.2	85	
11	1	1	6.2	28.6	21.7	112	
12	1	1	36.6	21.6	169.4	125	
13	1	1	38.5	35.5	108.5	71	
14	1	1	10	31.8	31.4	73	
15	0	1	0.4	19.1	2.1	113	
16	1	1	7.2	30.9	23.3	71	
17	1	1	14.1	14.7	95.9	82	
18	1	1	14	25.4	55.1	106	
19	0	0	0	24.7	0.0	49	
20	0	0	1.7	49.4	3.4	31	
21	0	0	0	47.7	0.0	84	
22	0	1	28.9	13.2	218.9	26	Slough
23	0	0	0.7	35.6	2.0	67	
24	0	0	0	12.1	0.0	74	
25	0	0	3.2	46.3	6.9	77	
26	0	1	21.1	4.9	430.6	52	Slough
27	1	1	3.8	30.1	12.6	116	

**Table 2:** The result of determination of necrosis (*n* = 27) with medical specialists vs. image identification algorithm.

		Diagnosed by Image Identification Algorithm	
		With Necrosis	No Necrosis
Diagnosed by medical specialists	With necrosis	14	3
	No necrosis	0	10 *

* Including two cases with slough. Fisher’s exact test, *p* = 3.39 × 10^−5^.

**Table 3:** The result of determination of necrosis (*n* = 27) with medical specialists vs. image identification algorithm with the black-white pixel ratio.

		Diagnosed by Image Identification Algorithm	
		With Necrosis	No Necrosis
Diagnosed by medical specialists	With necrosis	17	0
	No necrosis	0	10 *

* Including two cases with slough. Fisher’s exact test, *p* = 1.19 × 10^−7^.

**Table 4:** The result of determination of wound depth (*n* = 27) with medical specialists vs. image identification algorithm.

		Diagnosed by Image Identification Algorithm	
		With Necrosis	No Necrosis
Diagnosed by medical specialists	With necrosis	20	0
	No necrosis	0	7

Fisher’s exact test, *p* = 1.13 × 10^−6.^

**Table 5:** Data of 23 additional cases with decubitus ulcers.

Case	Necrosis	Depth	Black (%)	White (%)	Black/White	Red	Comment
28	0	0	0.1	34	0.3	60	
29	0	0	0	36.4	0.0	134	
30	1	1	27.6	35.7	77.3	108	
31	0	1	2.5	42.9	5.8	115	
32	0	1	1.5	45.2	3.3	108	
33	1	1	29.4	45.3	64.9	80	
34	1	1	21.4	40.6	52.7	90	
35	0	1	4	2.1	190.5	120	
36	1	1	25.9	16.5	157.0	114	
37	1	1	14	26.6	52.6	109	
38	1	1	12.9	35.9	35.9	90	
39	1	1	5.9	42.4	13.9	104	
40	1	1	8.1	42.6	19.0	125	
41	0	1	2.3	17	13.5	131	
42(1)	1	1	22	44.8	49.1	108	
42(2)	1	0	18.8	17.5	107.4	46	Necrosis (partial)
43	0	0	1.1	26.5	4.2	76	
44	1	1	38.6	43.6	88.5	75	
45	1	1	29	49.4	58.7	106	
46	0	0	0	29.4	0.0	39	
47	0	0	3.9	39	10.0	45	
48	0	0	3.6	38.3	9.4	78	
49	1	1	14.9	38.9	38.3	86	
50	1	0	21.1	21.1	100.0	44	Necrosis (partial)

**Table 6:** The validation of determination of necrosis with medical specialists vs. image identification algorithm.

		Diagnosed by Image Identification Algorithm	
		With Necrosis	No Necrosis
Diagnosed by medical specialists	With necrosis	14	0
	No necrosis	0	10

Fisher’s exact test, *p* = 5.10 × 10^−7^.

**Table 7:** The validation of determination of wound depth with medical specialists vs. image identification algorithm.

		Diagnosed by Image Identification Algorithm	
		With Necrosis	No Necrosis
Diagnosed by medical specialists	With necrosis	17	0
	No necrosis	0	7

Fisher’s exact test; *p* = 2.89 × 10^−6^.

## Data Availability

The data presented in this study are available upon request from the corresponding author.
